# Hotspots 2.0: Toward an integrated understanding of stressors and response options

**DOI:** 10.1007/s13280-018-1120-1

**Published:** 2018-11-20

**Authors:** Ahmed S. Khan, Georgina Cundill

**Affiliations:** 1 International Development Research Centre, Ottawa, K1P 0B2 Canada; 2 Present Address: African Development Bank Group, Abidjan, Côte d’Ivoire

**Keywords:** Biodiversity conservation, Climate adaptation, Hotspots 2.0, Human development, Policy integration, Social and ecological resilience

## Abstract

Integrated and transdisciplinary approaches are necessary in hotspots research where the intention is to influence policy and practice. Knowing that climate change will impact major ecosystem services and the sustainability of life support systems, a critical examination of the hotspot concept and approach is undertaken to pursue synergistic responses. Hotspots 2.0 embodies current thinking about planning towards multiple drivers of change and seeing human and natural systems as mutually inter-dependent and benefiting from integrated policy approaches. Such proposed adaptation interventions to inter-related stressors will complement biodiversity conservation, disaster risk reduction, and human well-being. Through a systematic review, we assess 114 relevant peer review cases to examine integrative responses to climatic and non-climatic vulnerabilities in various hotspot regions. Furthermore, we illustrate the utility of the Hotspots 2.0 approach using emerging insights from the ‘Collaborative Adaptation Research Initiative in Africa and Asia’ in semi-arid regions, deltas, and glacier-fed river basin hotspots.

**Electronic supplementary material:**

The online version of this article (10.1007/s13280-018-1120-1) contains supplementary material, which is available to authorized users.

## Introduction

Responses to climate change and other sustainable development goals (SDGs) will require holistic approaches that simultaneously deal with major environmental and social pressures that threaten social-ecological systems (SES). Here, we employ the concept of Hotspots 2.0 as a tool to identify multiple stressors and response options in areas of the world most vulnerable to climate change and therefore in need of integrated response options. The hotspot concept offers an entry point for identifying and assessing such integrated responses in SES. While the concept originated in conservation science in the late 1980s with a focus on species extinction and endemism (Myers et al. 2000), it has become a useful tool to address diverse social concerns including disaster risk reduction (DRR), food security, and resilience building (Hare et al. 2011; Fraser et al. 2013; De Souza et al. 2015). From a planning standpoint, Hotspots 2.0 are regions with some combination of strong climate signals, fragile and sensitive ecosystem services, resource-dependent livelihoods, vulnerable human populations and limited adaptive capacity to cope with multiple stressors (Giorgi 2006; Bellard et al. 2014; de Sherbinin et al. 2015; De Souza et al. 2015). Three events have changed the way hotspots are perceived and operationalized in policy and practice. The first turning point was the 1992 Rio summit that strengthened linkages between conservation and development through multilateral and global conventions on biodiversity, desertification, and climate change. This nurtured institutional mandates and specific management responsibilities towards specific hotspot regions such as mountains, semi-arid areas, coastal regions, and tropical forests. The second decisive moment was the Johannesburg 2002 Summit, which brought together various stakeholders to develop sustainability strategies for current and future generations. The SDGs are a continuation of these initiatives and include amongst others, efforts towards zero hunger (SDG 2), gender equality (SDG 5) climate action (SDG 13), and biodiversity conservation (SDG 14 & 15). The final episode that reshaped our understanding of hotspots and that highlighted the need for integrated responses to vulnerability is natural disasters such as the 2004 Asian Tsunami, the Szechuan earthquake, Hurricane Katrina and others that required billions of dollars for emergency responses and disaster relief.

These past developments prompt a reflection on salient questions regarding monitoring climate policy integration with DRR and human development especially the SDGs (Szabo et al. 2016). Although there is a common understanding to embrace multiple drivers of change (including but not restricted to climatic drivers), there remains a paucity of analytical frameworks and research-support mechanisms to promote such integrative thinking and action. How and when climatic and non-climatic drivers interact to impact ecosystems and human well-being is a concern to both researchers and policy makers. Moreover, where (and with whom) can we identify entry points and policy windows into these complex social-ecological interactions and governing spaces present opportunities for synergy and partnerships.

Through a systematic review on ‘hotspots’ and related vulnerability terms using bibliometric techniques, we assess the usefulness of the Hotspots 2.0 concept to address multiple drivers of change and to seek entry points for policy integration and coherence. We begin with framing climate hotspots as coupled SES, under multiple stressors and vulnerabilities, with various policy integration windows for human development, conservation planning, and social-ecological resilience. Through an integrated assessment of drivers and impacts in major hotspots, we synthesize integrated policy responses of relevance to policy and practice. Lastly, we illustrate the utility of this approach through research synthesis activities in a major research consortium where the hotspot concept has been a central feature in addressing adaptation planning challenges. We conclude with insights for future transdisciplinary research on hotspots and policy implications for practitioners and researchers. We underscore how integrated responses to climatic and non-climatic drivers of change can offer complementary policy directives to support National Adaptation Plans (NAPs) and attaining the SDGs.

## Materials and methods

### Framing hotspots as social-ecological systems under multiple threats

**Fig. 1:**
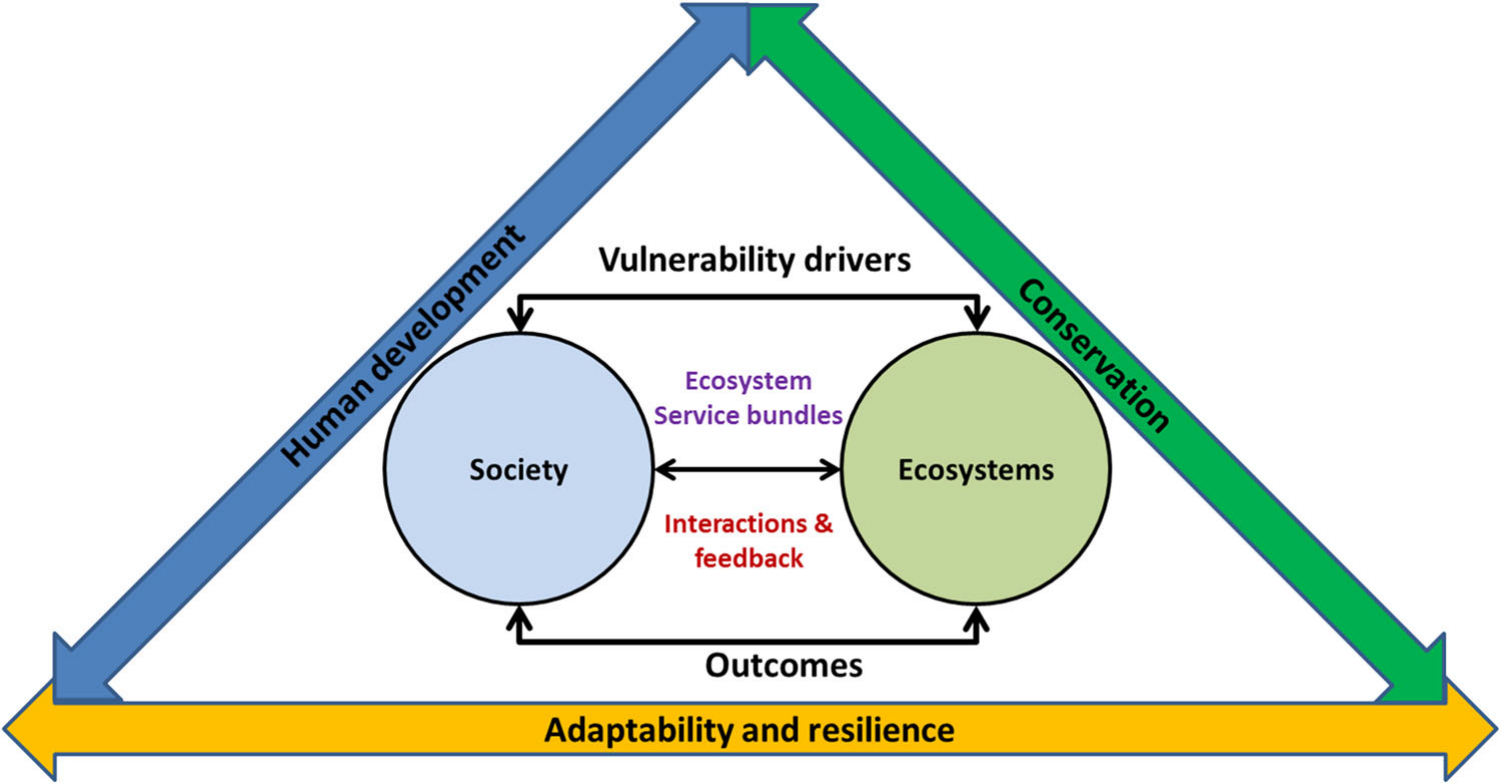
Hotspots 2.0 as coupled SES with multiple threats and policy integration windows (Adapted from Bennett et al. 2009; Khan et al. 2018)

Whatever framing is used to identify hotspots, these areas are understood as complex SES that are impacted by multiple drivers and stressors. As in all coupled and complex systems, hotspots provide multiple ecosystem services, which vary across space and time in terms of natural attributes, societal demands, and corresponding feedback (MA 2005; Liu et al. 2007a; Ostrom 2009). Figure 1 is a conceptual framework that can support such an interdisciplinary approach for developing integrated response strategies in diverse hotspots settings. Indeed, the urgency of response will depend on the fragility and sensitivity of ecosystems, the quantity and quality of resources they provide (both extractive and non-extractive), and the rules and norms that govern human activities (Berkes and Folke 1998; Ostrom 2009). SES approach to hotspots provides opportunities to identify and assesses entry points towards policy integration as evident in land use cover change (LUCC) or coastal deforestation (Mantyka-Pringle et al. 2015; Ramesh et al. 2015).

In glacier mountain hotspots, for example, synergistic and resilient outcomes may require attention to the cryosphere as well as hydrological dynamics with regard to melt water, erosion controls, and human welfare (Wramneby et al. 2010; Lutz et al. 2016). Building resilience (both social and ecological resilience) through strategic interventions that accentuate desirable feedbacks are critical. As shown in Fig. 1, successful outcomes will then require meeting integrated policy objectives relating to conservation, resilience, and development. In this case, integrated resilient development outcomes will entail societal well-being, gender-sensitive livelihoods, and sustainable production and consumption (Kilroy 2015). Similarly, ecosystem-based adaptation (EbA) outcomes will be influenced by restoration as well as land use planning that mitigates flooding impacts (Khan and Amelie 2015).

Matching the scale of decision-making frameworks to the scale of drivers and impact presents a challenge in social-ecological systems (Platts et al. 2013). Whilst place-based interventions (e.g., zoning by-laws) and sectoral adaptation issues (e.g., climate smart agriculture) may fall within NAPs, transboundary responses often fall outside of national jurisdictional mandates and call for regional approaches. Often, these regional challenges are championed through networks of scientists and practitioners that rely on long-term global change research in hotspots (Hobday et al. 2016). In semi-arid hotspots in Africa, for example, we are now seeing new developments towards regional scientific networks such as the Sahel and Sahara Observatory that monitors agro-climatic risks and contributes to development issues at multiple scales (Yevide et al. 2016). Although these findings suggest increasing trends towards broader geographical scales and more integrated approaches, there have been limited options and opportunities for regional-level responses to multiple drivers of change. Certainly, transdisciplinary research that explores integrated approaches to dealing with global change is imperative in addressing future climate hazards. How and where can policy interventions be leveraged in addressing multiple drivers of change? And with whom can cross-scale initiatives and partnerships be developed? These are some of the salient questions explored in the systematic review below.

### Conducting systematic review

#### Search strategy and bibliometric analysis

We employ bibliometric tools to identify and select relevant case studies that characterized hotspots in multiple biomes. Through a three-step integrated assessment approach, we started with a keyword search strategy in several journal databases including Web of Science (WoS), Scopus and EBSCO to identify refereed articles from the period 1988. This is when the hotspot concept was coined by Norman Myers and became a conceptual tool for conservation planning. The search terms comprised of a combination of thematic issues, policy entry points, and approaches to multiple drivers of change. The search included four tiers: [“climate” or “biodiversity” or “vulnerability” or “adaptation” or “disaster risks”] AND [“hotspots”] AND [“conservation” or “adaptation” or “resilience” or “land use change”] AND [“population” or “food security” or “governance” or “poverty”]. We used Boolean connectors “AND” to peruse the search results and after limited success we used “OR” to increase the literature outputs in each tier. Using the WoS categorization scheme, we found 509 (36%) cases on Ecology, 342 (24%) on Biodiversity Conservation, 320 (23%) on Environmental Sciences, 120 (9%) on Evolutionary Biology, and 119 (8%) on Multidisciplinary Studies (mostly meteorology and atmospheric sciences). Of the document types, journal articles made up 90% of outputs. The remaining 10% comprised of reviews (6%), proceedings (3%), and edited book chapters (1%). We focus mostly on journal articles and excluded non-peer reviewed technical reports and government documents.

#### Screening and inclusion criteria

**Fig. 2:**
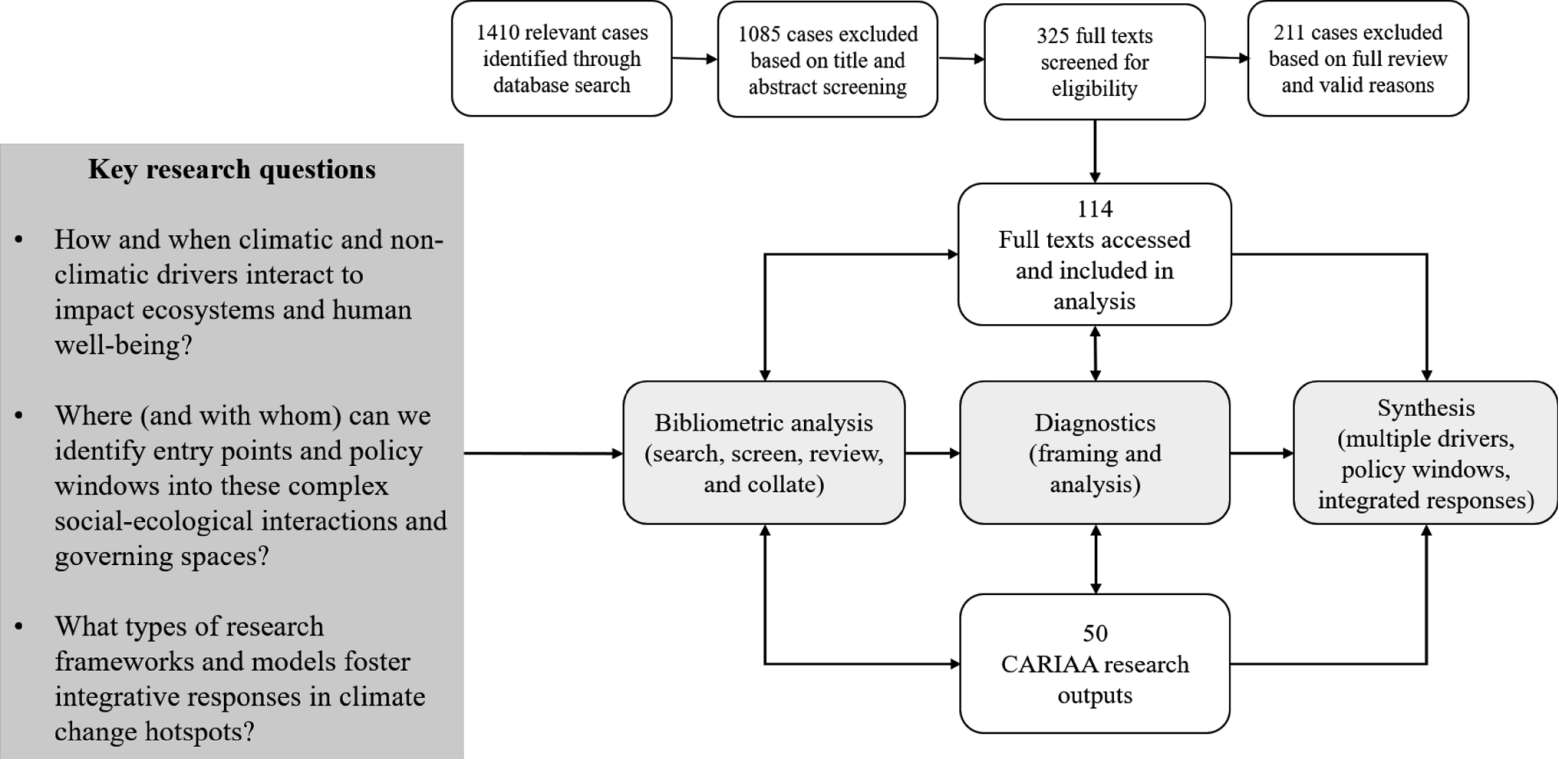
Schematic steps and approaches for the integrated assessment and synthesis

The second step was to screen the title and abstracts as well as the keywords to see if the hotspot embraced two or more of the related fields. Out of the initial 1410 search results generated, 1085 cases were excluded owing to limited disciplinary relevance and poor hotspot interpretations. In addition, 325 full texts were eligible and screened for the analysis. We further excluded 211 papers in cases where they did not meet our inclusion criteria (Fig. 2). For example, we excluded articles that did not deal meaningfully with hotspots or climate stressors, even in cases where they addressed vulnerabilities and human development concerns (see for example Kok et al. 2010). A transdisciplinary use of the hotspot concept beyond conservation and DRR (and consideration to climate stressors) was a major criterion for inclusion in the assessment. The rationale behind the inclusion criteria for Hotspots 2.0 was to capture both climatic and non-climatic drivers, their interactions, and integrated responses (O’Brien and Leichenko 2000). This approach is consistent with the PRISMA^1^ framework in terms of inclusion and exclusion criteria. Only 114 articles were included in the final analyses (see Fig. S1, Electronic Supplementary Material). The Driver-Pressure-State-Impact-Response (DPSIR) framework was used to synthesize these variables (Gari et al. 2015), which has the unique feature to identify and categorize multiple interacting drivers and to link feedbacks with institutional responses (Newton and Weichselgartner 2014).

#### Diagnostics, categorization and synthesis

We collated and synthesized data for major hotspots as shown in Table S1 using the DPSIR approach. In understanding system properties, we used a diagnostic tool that entails probing questions relating to how diverse, complex, dynamic, multi-scaled, and sensitive are hotspots. The five major hotspots categories pertinent to our assessment include (i) alpine and glacier-fed mountains, (ii) arid and semi-arid regions, (iii) estuaries and river deltas, (iv) deciduous and tropical rainforest, and (v) marine and coastal seascapes (including Small Island Developing States). This classification is not exhaustive nor limiting, but rather exemplifies our concept of Hotspots 2.0 as SES that exemplify strong climate signals, fragile and sensitive ecosystem services, resource-dependent communities and limited adaptive capacity. Urban regions and landscape approach were not considered separately but inherently as part of ecosystem interactions, although key drivers of change such as ‘population’ and ‘urbanization’ were underscored. The rationale for the above typology was to integrate Norman Myers—Conservation International biodiversity hotspot characteristics (e.g., endemism and species extinction risks), the Köppen Climate classification scheme paying attention to biogeographical attributes and climate stressors, and fragile or sensitive ecosystems as defined in Agenda 21. For consistency, the principle of ecological representativeness was very helpful for cross comparison of cases as well as in understanding the level of exposure, sensitivity, and adaptive capacity.

We further examined the contribution of a consortium-styled research partnership on climate hotspots, the Collaborative Adaptation Research Initiative in Africa and Asia (CARIAA). A seven-year donor funded program aimed at understanding regional challenges and pursuing solutions in three hotspot regions across Africa and Asia. Through their research outputs over 5 years, we identified multiple integrated adaptation responses. The responses targeted either key policy entry points in the social (e.g., nutritional well-being) or ecological systems (e.g., conservation). At the societal level, responses could be institutional and focus on nurturing the adaptive capacity of community leaders or women entrepreneurs in vulnerable households (Rao et al. 2017). Within ecosystems, interventions could target environmental planning, stewardship initiatives and conservation programs (Rasul and Sharma 2016). For successful outcomes (e.g., resilient communities and cities), decision-making frameworks are vital in addressing trade-offs, spatial planning tools, and user conflicts over resources (Karpouzoglou and Vij 2017). How and when windows of opportunities arise are critical to the type of integrated policy measures developed and the nature of stakeholder partnerships.

**Fig. 3:**
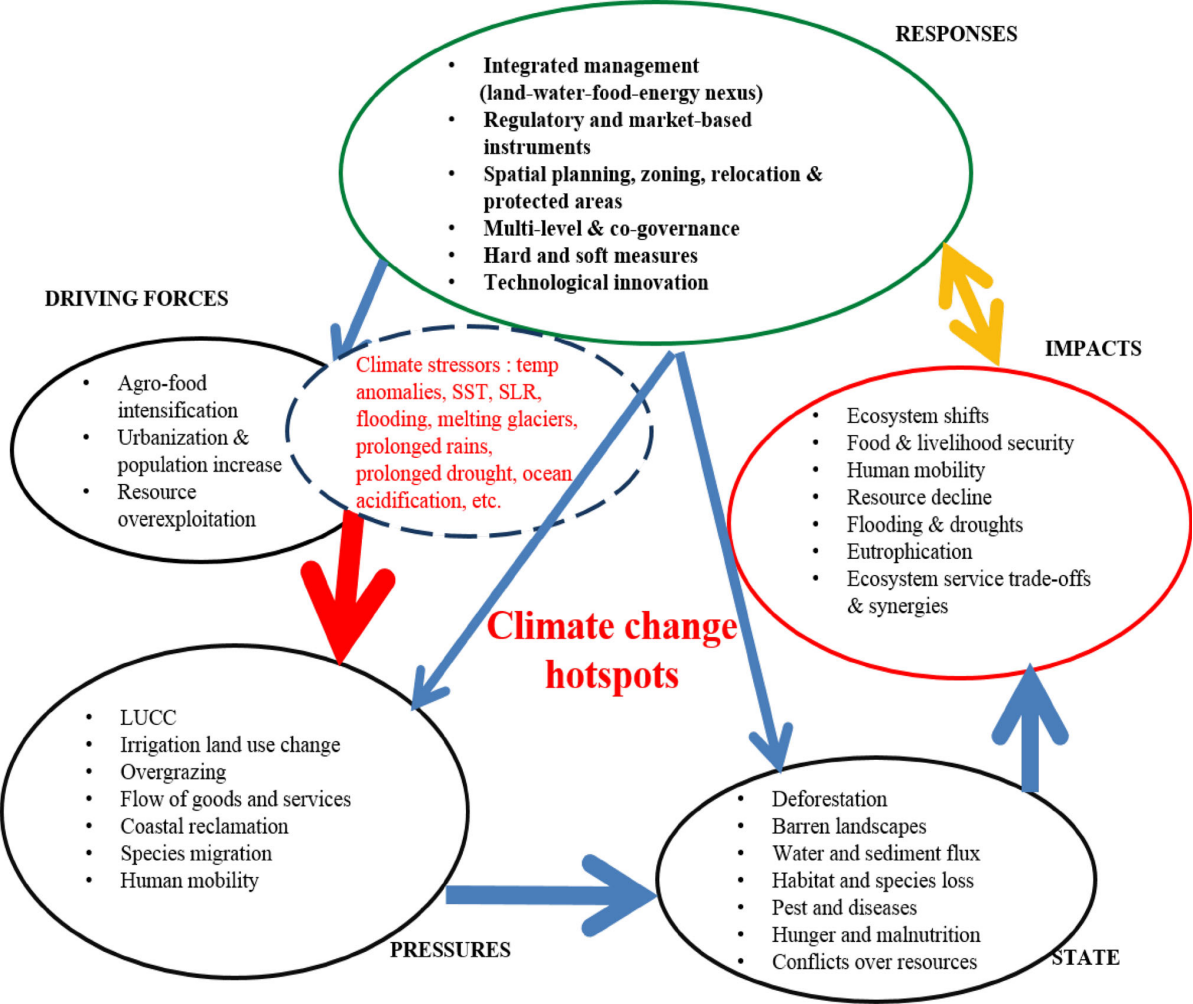
Synthesis of drivers, impacts, and responses in some illustrative hotspots

Finally, through infographics and charts, the multiple threats, impacts and integrated responses are synthesized to demonstrate synergistic responses in hotspots regions. The syntheses using the DPSIR framework are presented in Fig. 3 and Table S2. Figure 6 is a map that sums up various kinds of integrated responses in 14 countries using CARIAA research outputs.

## Results and discussion

**Fig. 4:**
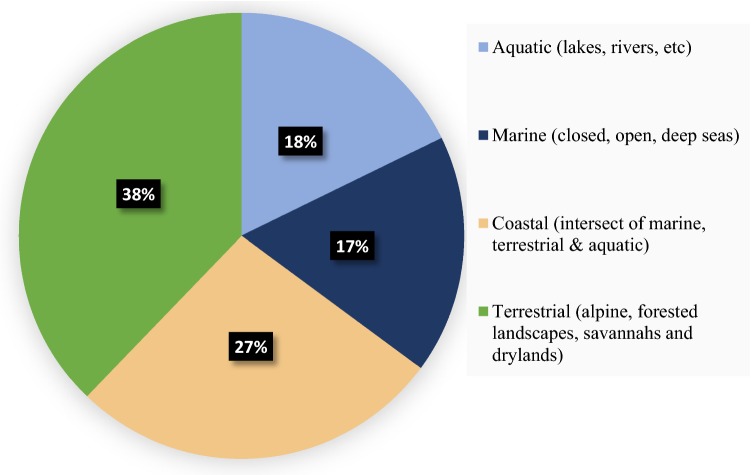
Climate hotspots research in various biomes and ecoregions

A wide variety of global change drivers, pressures, and impacts were evident in hotspots as shown in Fig. 3. As in the case of most complex systems, and this is particularly true of Hotspot 2.0, both anthropogenic and non-anthropogenic drivers interact at various scales to impact and change living systems and the built environment. From our database of 114 cases assessed, more than one-third of hotspots are in terrestrial biomes and eco-regions. These include alpine, forested landscapes, savannahs, and dryland regions (38%). Next in occurrence are estuaries, deltas, and coastal systems (27%), followed by freshwater including lakes (18%), and marine realms (17%) comprising of both open and closed seas and coral reefs (Fig. 4). With multiple interacting drivers and pressures affecting hotspots, some stressors and impacts are quite unique and context specific. For example, alpine and glacier-fed mountains are highly vulnerable to climate stressors that impact ice cover and stream flow in major river basins especially on the Asian continent. In effect, melting glaciers due to increasing temperatures could trigger flood risks and runoffs that threaten human settlements and major livelihood activities such as agriculture downstream (Wassmann et al. 2009). Feedback and response mechanisms in such contexts involved several sectors, institutions, and stakeholders across boundaries (Khamis et al. 2014).

Similarly, in riparian ecosystems and major river deltas, hydrological fluctuations triggered by upstream precipitation affect fluvial geomorphology and various supporting and regulating ecosystem services (Davies 2010; Vermaat and Eleveld 2013). In semi-arid regions, in contrast, extreme temperatures cause water stress and impact food production and regional economic development (Liu et al. 2008; Fraser et al. 2013). In offshore marine realms, ocean acidification is causing damage to coral reef ecosystems, altering species migration and affecting seafood supply for coastal communities (Descombes et al. 2015; Hobday et al. 2016). Given that the majority of the world population lives in coastal regions and deltas, with rapid demographic changes influencing critical habitats and agro-food systems, global change strategies need to consider various adaptation pathways (Hugo 2011; Hermans-Neumann et al. 2017). Spatial planning responses are imperative to this effect owing to raw material demand that accelerates land use cover change and environmental degradation (Wetzel et al. 2012; Hauer 2017). Moreover, managed retreat has been recommended as an adaptation option to flooding hazards (Hino et al. 2017), although there are gaps in developing the institutional mechanisms towards social and ecological resilience in migrating and resettling areas (Wetzel et al. 2012). Synergistic responses through ecosystems-based adaptation and marine spatial planning have worked well in addressing multiple drivers of change on vulnerable coastlines particularly in Small Island Developing States. In Papua New Guinea, for instance, ‘climate refugia’ is used as a management tool to integrate biodiversity into National Adaptation Plans (Game et al. 2011).

**Fig. 5:**
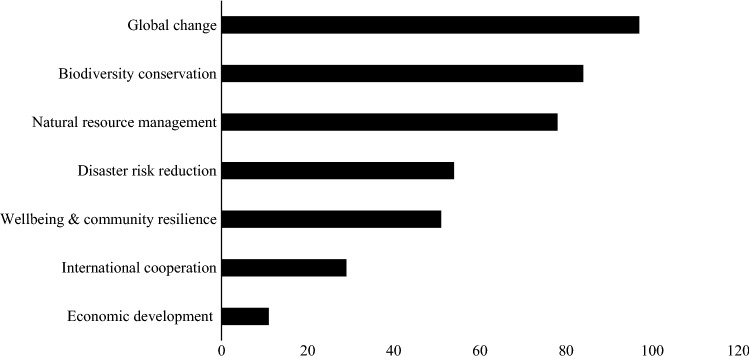
Thematic entry points to climate policy integration (n = no. of cases)

The notion that climatic and non-climatic drivers interact on multiple levels and often impact life support systems is not new (MA 2005). Yet, addressing these impacts in the developing world can be challenging particularly where transformational outcomes are needed but institutional capacity is low (Colloff et al. 2017). In addition, joint responses that address climatic and development priorities require an enabling environment for inclusive and multi-level governance arrangements (Hannah et al. 2013). The Hotspot 2.0 concept offers a planning tool and an entry point to prioritize and integrate policy responses in SES contexts, while appreciating the complex and dynamic linkages among multiple stressors. In all the cases assessed, four major entry points were identified for developing integrated response strategies: (i) global strategies towards adaptation and mitigation, (ii) the sustainability of natural resource management and biodiversity conservation, (iii) natural hazards and disaster risk, and (iv) human well-being and international cooperation (Fig. 5).

The integrated response strategies often take many forms through various entry points that include fiscal instruments, hard and soft measures, internet and communication technologies (ICTs) such as in early warning signals, and multi-level governance arrangements. Governance is central to integrative approaches with full stakeholder involvement regarding compliance and stewardship (Khan et al. 2018). As such, conservation priorities and development planning are two of many windows of opportunities to mainstream climate interventions especially at the synergistic interface of ecosystem-based adaptation and resilient development. From a multi-scale and cross-scale approach, about one-third (33%) of all the cases assessed were at the local and sub-national level, with the majority at the regional and global level (64%) and a small fraction nested at multiple scales. Such multi-scale research demonstrates the growing interests in interdisciplinary approaches, research partnerships, broad readership, as well as multiple co-authorships (Figs. S1–S5).

## Insights from the CARIAA program on climate change hotspots

**Fig. 6:**
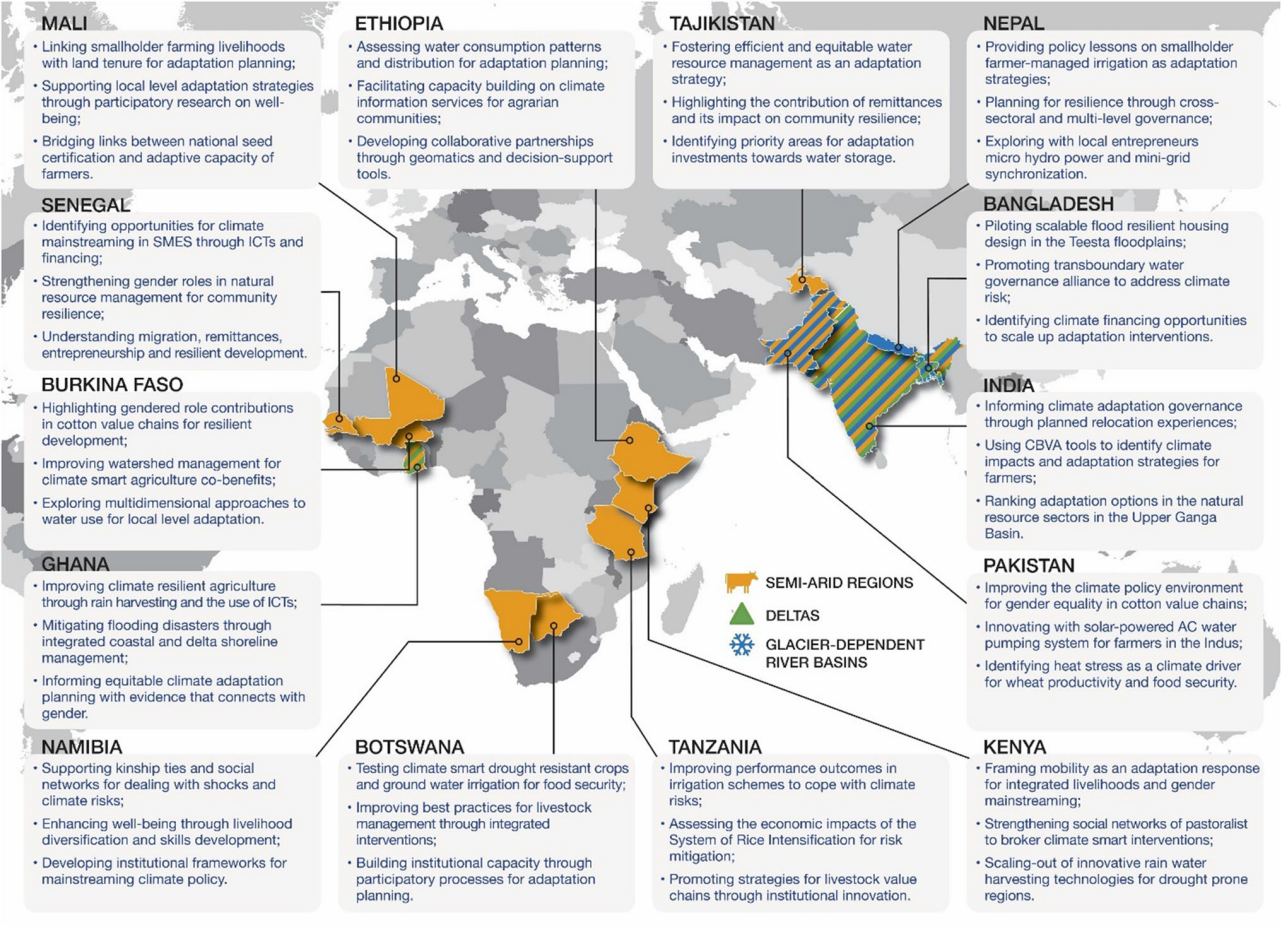
Key emerging insights on integrated responses in meeting SDGs from CARIAA

Climate change undermines the sustainable use of natural resources and human well-being especially for the most vulnerable regions and communities. It is therefore critical to support local and national strategies in regions where adaptive capacity is low and human development needs are high. This has been the motivation behind CARIAA, pursued through a network of four research consortia that bring together partners from academic institutions, government, civil society, and the private sector. The goal is to support the production of new knowledge, share expertise, and inform policy and practice. More than 450 researchers and practitioners are involved in the three hotspots, with 40 implementing partners, and 18 core institutions in more than 14 countries (Cochrane et al. 2017). As shown in Fig. 6, various policy entry points were targeted in multiple domains. For example: (i) conservation priorities as entry points for land-use planning and watershed management in Burkina Faso; (ii) livelihood diversification for community resilience in Namibia; (iii) planned relocation from highly vulnerable regions as adaptation strategies such as in Kenya and India; (iv) the use of rain harvesting technologies and ICTs in Ghana and Kenya; (v) gendered value chain developments for cotton production in Pakistan; and (vi) scaling-up of small-holder irrigated farming in Nepal.

Although these responses vary across countries and regions, some common features can be identified especially in creating partnerships to influence policy outcomes relating to desertification, biodiversity and climate change. For instance, distinct from the traditional partnerships between NGOs and donors towards conservation or development planning, there is an increasing involvement of individuals and private sector players working on agrarian livelihoods, loss and damage, and financing mechanisms (Vincent et al. 2016). Equally so, donor funding that support consortium research is fundamental in creating partnership and communities of practice. It also contributes to building an evidence base to show how, where and when stakeholders take the initiative in creating spaces for climate policy formulation and development activities.

In Kenya for example, livestock herders and small-medium enterprises in Maasai Mara are building resilience through integrated livelihoods that builds on on-farm activities such as eco-tourism (Bedelian and Ogutu 2017). Similarly, policy coherence and multi-level decision-making structures that nurture social norms and empower local traditional governors in agricultural practices can be successful in the light of climate stressors in Mali (Biemans et al. 2016). In the Himalaya mountains of Nepal, the transboundary nature of water governance has motivated researchers to seek institutional partnership and facilitate water treaties (Rasul 2014; Arfanuzzaman and Syed 2017). In low-lying coastal countries like Bangladesh, integrated modeling of biophysical and social realms are contributing to national delta management plans and coastal resilience (Welch et al. 2017). These collaborative partnerships and entry points for policy uptake are increasing important in creating long-term relationships towards communities of practice and research networks for responding to future hazards.

## Implications for policy and practice

The hotspot concept is a useful analytical tool for integrating multiple response strategies towards global change drivers and in meeting sustainability targets. Unlike earlier hotspot approaches that focus on a specific type of vulnerability such as biodiversity loss or natural disasters, Hotspots 2.0 offer researchers and decision-makers multiple entry points to explore interacting threats to climatic and non-climatic drivers of change through complementary responses. They also provide opportunities for cross-scale learning on climate-related hazards and to explore transboundary governance issues through research networks and communities of practice at multiple levels (Liu et al. 2007b; Cundill et al. 2018).

Because future climate change will affect all aspects of human activities (e.g., food production, commodity supply chains, essential infrastructure, and critical ecological functions), attention to both the social and natural realms is crucial for building adaptive capacity and exploring resilient outcomes. Identifying appropriate entry points (and stakeholder groups) for climate policy integration into conservation or development planning is one of many ways of bridging the science policy interface and in empowering local champions and attaining social-ecological resilience. Although most climate policy interventions (such as NAPs) tend to be separate and disconnected from many other sustainable development interventions (e.g., gender mainstreaming or decentralization), there is scope to explore policy windows and leverage points for cross-sectoral partnerships. By providing a holistic view of vulnerability, stressors and responses, a hotspot approach can support efforts to integrate broader climate policy imperatives into localized and national economic planning.

## Electronic supplementary material

Below is the link to the electronic supplementary material.

Supplementary material 1 (pdf 688 kb)

## Footnotes

1 PRISMA: Preferred Reporting Items for Systematic Reviews and Meta-analyses.
