# Supplementary material for: Hotspots 2.0: Toward an integrated understanding of stressors and response options
Source: Ambio. 2018 Nov 20;48(6):639–48. doi: 10.1007/s13280-018-1120-1 (PMC6486930; doi:10.1007/s13280-018-1120-1)
Supplement: Supplementary file 1 — Supplementary material 1 (pdf 688 kb) [file 13280_2018_1120_MOESM1_ESM.pdf]

AMBIO

ELECTRONIC SUPPLEMENTARY INFORMATION

This supplementary material has not been peer reviewed

Title: **Hotspots 2.0: Toward an integrated understanding of stressors and response options**

Authors: Ahmed S Khan and Georgina Cundill

Content description

Rubric for data collection

Table S1 Diagnostic tool for case study assessments

Fig. S1 Spatial scale of research activities (no. of cases)

Fig. S2 Interdisciplinary research methods and approaches evident in cases (n=114)

Fig. S3 Institutional affiliations in hotspot 2.0 research

Fig. S4 Top journals that published climate related hotspot research

Figure S5 Country of 1st authorship and amount of outputs

Table S2 Synthesis from selected full text review on hotspots using the DPSIR framework

References included in systematic review

CARIAA Research outputs included in synthesis

Content description

For the 114 hotspot cases identified and relevant for the analysis (Figure 2), a database was created with key information obtained on: year of publication, geographical region, scale of research, methodological approaches, author affiliations, journal types, donor agencies, disciplinary strands, and policy entry points. The rubric and diagnostic tool is provided below as Table S1.

DATA EXTRACTION TOOL

1. Author(s)

2. Year

3. Journal

4. Country (1st author)

5. Funders

6. Affiliations

Academia	Private	Consulting	Public	Multilaterals	NGO

7. Scale

Local/ sub nation	National	Regional	Global

8. Geographical focus

Africa: Eastern Africa / Central Africa / Northern Africa/Southern Africa/ Western Africa
Americas: Caribbean/ Central America / South America / North America
Asia: Central Asia/ Eastern Asia / Southern Asia / SE Asia / Western Asia
Europe: Eastern Europe / Northern Europe / Southern Europe / Western Europe
Oceania: Australia and New Zealand / Melanesia / Micronesia / Polynesia

9. Conceptual framing

Methodological approach- climatic risk modeling or hotspot hazard mapping	Planning tool- conservation, urban or disaster risk planning	Place-based evidence- on eco-region e.g. Himalayas mountains or coral reefs

10. Methodological approaches

Field biophysical	Field social science	Integrated Field work	Climatology / Meteorology	Simulation Modeling	Spatial planning / Mapping	Experimental	Other

11. Transdisciplinary dimensions

Natural Resource Management	Conservation science	DRR	Global change	Well-being & community planning	Int'l Development /Cooperation	Macroeconomic development	Other

12. Biomes and eco-regions

Terrestrial	Aquatic	Marine
1-Mountain	5-lakes and ponds	8-open seas
2-Forest	6-rivers and streams	9- corals and deep seas
3- Rangeland / savanna	7-deltas/estuaries/wetlands	10- enclosed seas
4-built settlement		

Table S1: Diagnostic tool and rubric for integrated assessment and syntheses

	Natural Systems	Socioeconomic Systems	Governing System	Interactions & Feedbacks
Diversity	What are the types of species, and their biomass within the ecosystem?	What are the main ecosystem service co-benefits and beneficiaries?	What are the various mandates and policy framework to meet adaptation goals?	What institutional arrangements promote and limit performance outcomes?
	How do species interact: competition, mutualism, compensatory/depensatory processes?	How do rights and access influence compliance, stewardship, and response?	Who are the various organizations that influence policy design and implementation?	Are the adaptation policy instruments single or multiple objectives?
Complexity	What is the composition of the species and their richness? Endangered, endemic or keystone?	How do adaptation measures influence various groups: power, equity, and conflicts	What types of policy instruments can be are found within the ICZM and in adaptation toolkits?	What contextual factors promote or thwart adaptation interventions?
	How do factors such as currents, upwelling, dispersal, etc. affect ecological resilience?	Does adaptation policy instruments have a spillover effect to other sectors or regions?	Are there feedback mechanisms for policy re-formulation to seek funding?	What expectations from stakeholders affect adaptation outcomes?
Dynamics	What are the trends in resources appraisal and critical habitats during anomalies?	How do changes in macro- or micro-economic policies affect adaptation outcomes?	What structures or steering mechanism affects adaptation policy outcomes?	What are the trends in governance performance on adaptation?
	What short-term and long term (cyclical and non - cyclical) changes have taken place?	How will adaptation measures affect power brokerage, interest groups, and social networks?	How do changes in management vision or priorities affect success of adaptation intervention?	How often are the policy instruments monitored, evaluated, or re-designed?
Scale	Are LME considerations given for spatial processes and interactions across geo-political boundaries?	Does economic boundaries or cross-sectoral approach limit or enhance adaptation?	How do multi-governance structures affect the design and implementation of adaptation strategies?	Are there conflicts, non-compliance, or opposition to adaptation?
	Are these ecosystems and biophysical attributes unique, or representative: corals, eddy mixing, hotspots, etc.?	Does mobility of people affect ecosystem services and adaptive capacity?	How does the spatial scale of management influence institutional arrangements?	Are knowledge networks and systems integrated across space and time?
Sensitivity	What are natural or human-induced stresses, drivers, and threats that can be identified for ecosystem health?	What economic activities and livelihoods are most susceptible and their adaptation ranking schemes?	What precautionary and adaptive governing capacity are in place for climate change policies?	Are there warning systems, community resources for adaptive response?

Supplementary results Figures S1 to S5.

)

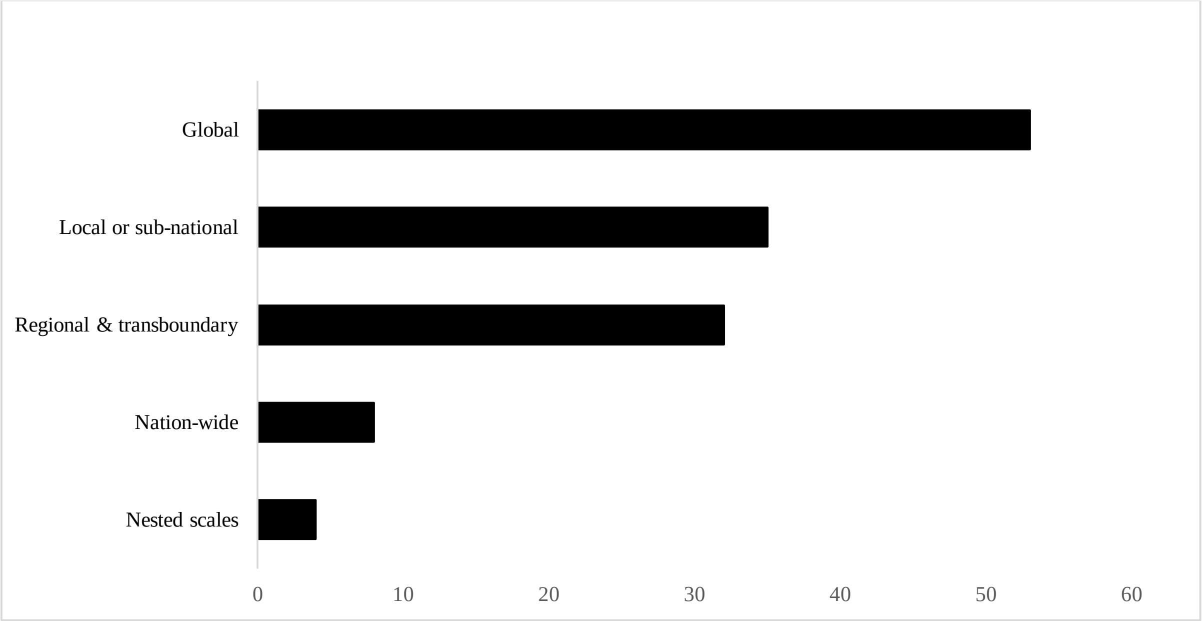

Fig. S1 Spatial scale of research activities (no. of cases)

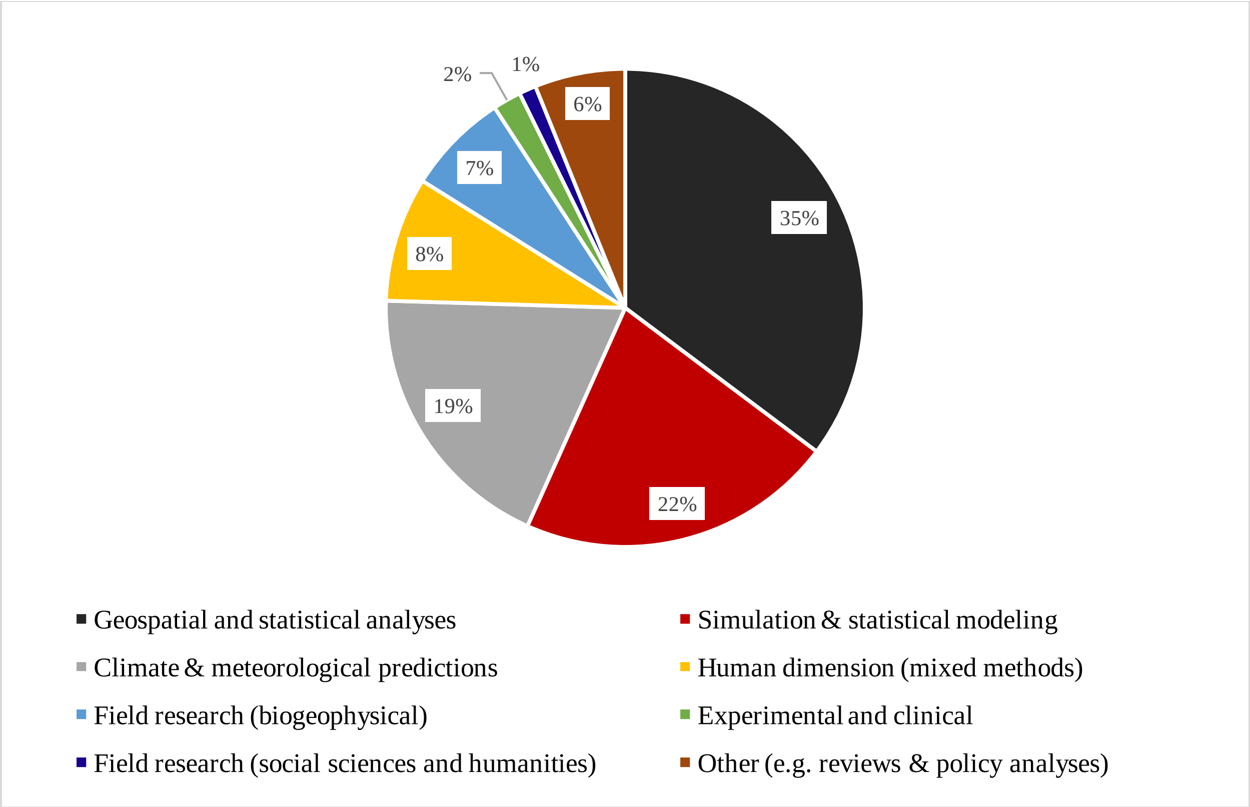

Fig. S2 Interdisciplinary methods and approaches in Hotspots 2.0 research (n=114)

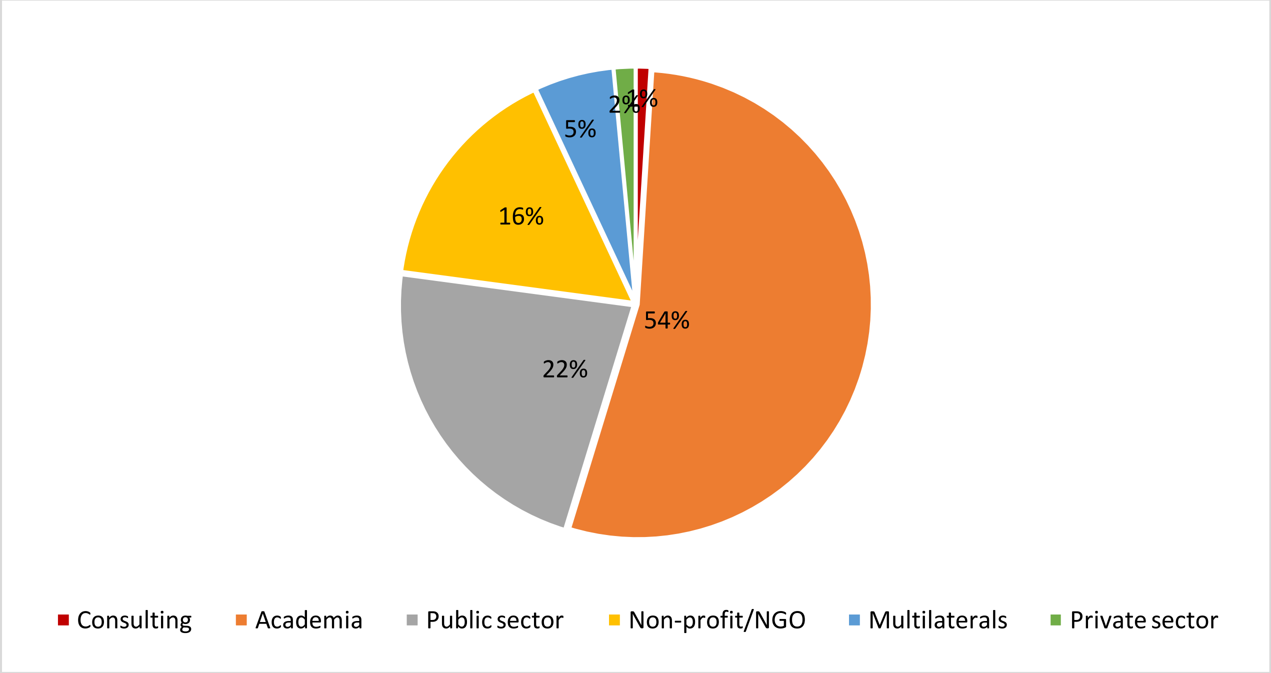

Fig. S3 Institutional affiliations in hotspot 2.0 research (n=114)

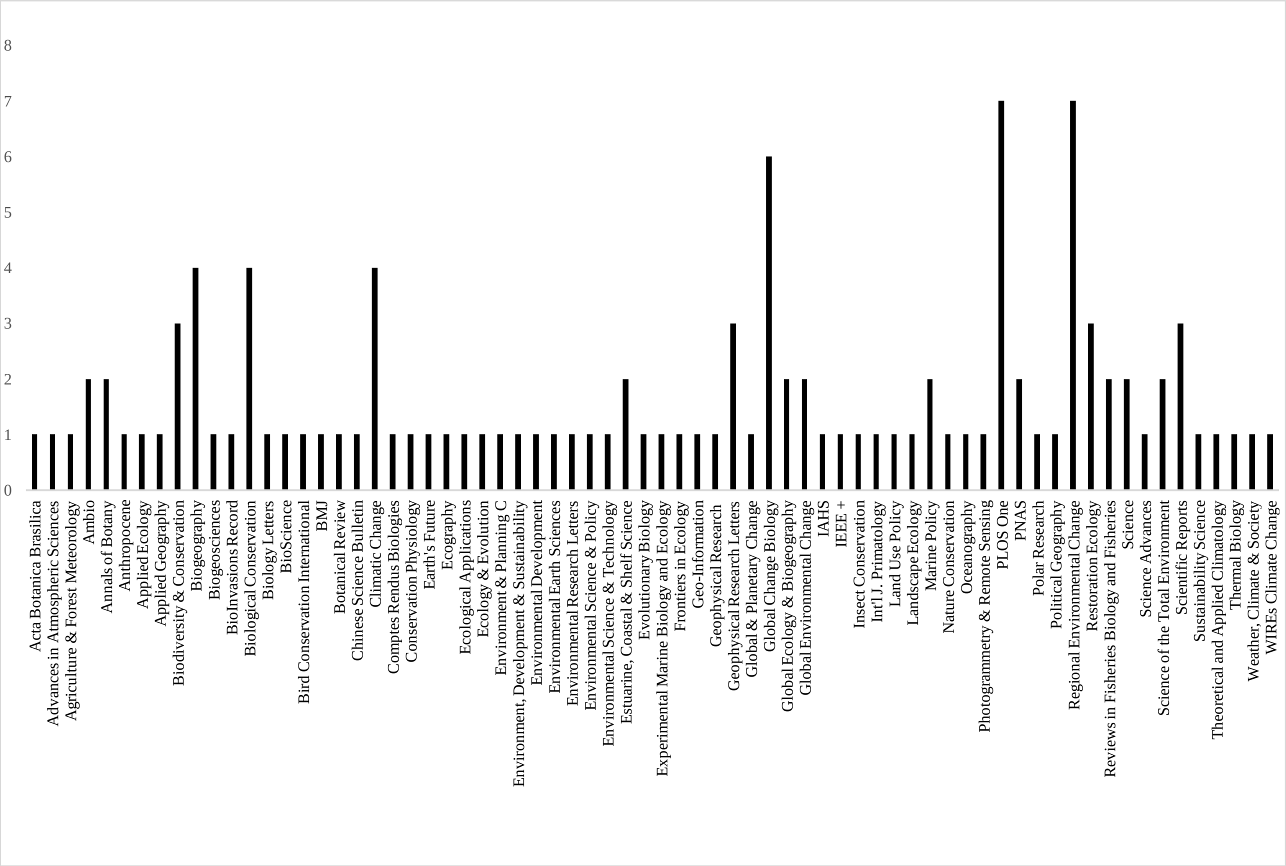

Fig. S4 Top journals that published climate related hotspot research

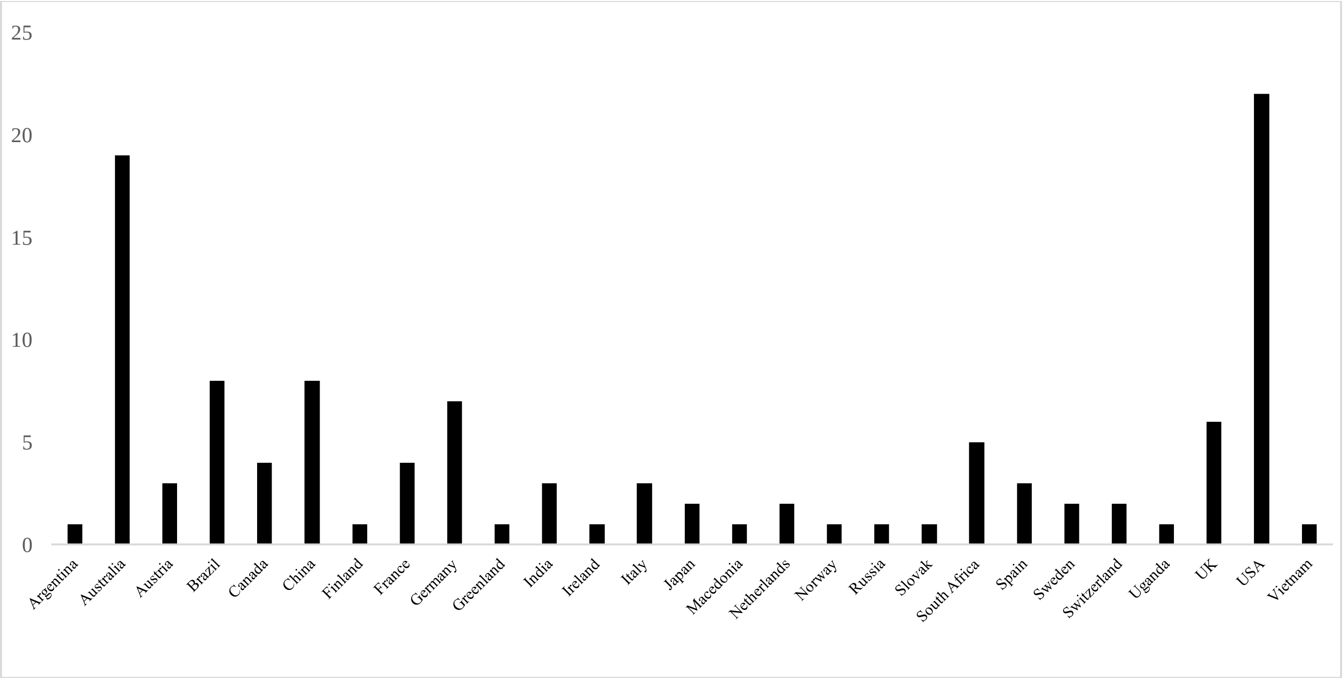

Fig. S5 Country of 1st authorship and amount of outputs

Table S2 Synthesis from selected full text review on hotspots using the DPSIR framework

	Drivers	Pressures	State	Impact	Responses	Cross-scale approaches
Alpine and glacier-fed mountains	Agro-forestry commodity prod	Habitat loss & extinction rates	Pest and diseases	Food & livelihood security	CBRM and NGOs inputs	International instruments
	Resource overexploitation	Landslides and LUC	Deforestation	Ecosystem shifts	Integrated NRM strategies	Regional management agencies,
	Urbanization and LUCC	High population density	Transportation networks	Human mobility	EIA, Standards, zoning & building codes	Social and trade networks
	Melting glaciers, stream flows & flooding				Institutional reforms, NAPAs capacity building	LTER, joint planning
	Energy	Dams & HEP	Water & sediment flux	Conflicts & displacement	Planned relocation / protected areas	Regional management agencies
Estuaries and deltas	Agro-food intensification	Irrigation and LUCC	Hydrological changes	Sediment run-offs	Integrated NRM strategies	Regional institutions
	Population density	Waste management	Eutrophication	Public & environ health	Environ planning & restoration	Social and trade networks
	Transportation	Flow of goods and services	Habitat loss and pollution	Water quality & species	Regulatory measure & standards	Regional management agencies
	Increasing rains and high stream flows	Farming and related human activities	Nutrient run-off, habitat and species loss, human migration	Flooding, eutrophication, human settlements	Relocation, migration, rezoning and resettlement	Transboundary water treaties, kinship ties and social and trade networks
Coastal and marine	Coastal development	Waste management	Eutrophication	Public health	Regulatory measures	Standards and monitoring
	Urbanization and pop clusters	Coastal reclamation & property dev	Habitat destruction	User right conflicts	Spatial planning and zoning	Social and trade networks
	Fisheries and marine resources development	Overcapacity / overexploitation	biomass and species loss	Resource decline	Management controls and various governance schemes	Regional agencies and int'l instruments
	SST & ocean acidification, SSL	conflict over access, increasing demand	dead zones, degrading ecosystems, low yield	fish migration, food insecurity, bankruptcy, etc.	MPAs, EbA thru coastal reclamation, PPP, hard measures	Spatial planning, RFMOs and bilateral management
Arid & semi-arid	Agro-food production	Water needs and LUCC	Soil infertility and LUCC	Food & livelihood security	Integrated agric prod (crops/soil)	Multilateral and regional collaboration
	Livestock management	Overgrazing	Barren landscapes	Desertification /lack of SOM	Restoration & techno innovation	Social and trade networks
	Household and industrial water consumption	H20 abstraction & hydrological flux	Poor water quality and quantity	Drought episodes	Tech innovation and rain harvesting	Bi-lateral and regional coop
	Poor rainfall patterns, strong winds and increasing temperature	Water access, quality and quantity.	Poor and loose soils, barren landscapes and poor crop yield	Conflicts between herders and farmers	Forecast & early warnings, CSA, incentives to innovate, water rights and pricing, irrigation, etc.	LTER, capacity building and knowledge transfer, partnerships
Deciduous and tropical rainforests	Timber & NTFPs	Loss in forest biomass	Deforestation	Shift in weather	Regulatory measures	Market based instruments
	Urbanization and growth	Housing development	LUCC	Human-wildlife	Integrated & multi-scale planning	Social and trade networks
	Energy and food prod	Agro-commodities	Loss of habitat & species	Ecosystem shifts	Restoration / afforestation	Standards and certification

References of papers included in the systematic review

Abermann, J., B. Hansen, M. Lund, S. Wacker, M. Karami, and J. Cappelen. 2017. Hotspots and key periods of Greenland climate change during the past six decades. *Ambio* 46. Springer Netherlands: 3–11. doi:10.1007/s13280-016-0861-y.

Asare-Kyei, D., F. G. Renaud, J. Kloos, Y. Walz, and J. Rhyner. 2017. Development and validation of risk profiles of West African rural communities facing multiple natural hazards. *Plos One* 12: e0171921. doi:10.1371/journal.pone.0171921.

Beatty, S. J., and D. L. Morgan. 2013. Introduced freshwater fishes in a global endemic hotspot and implications of habitat and climatic change. *BioInvasions Records* 2: 1–9. doi:10.3391/bir.2013.2.1.01.

Bellard, C., C. Leclerc, and F. Courchamp. 2014. Impact of sea level rise on the 10 insular biodiversity hotspots. *Global Ecology and Biogeography* 23: 203–212. doi:10.1111/geb.12093.

Bellard, C., C. Leclerc, B. Leroy, M. Bakkenes, S. Veloz, W. Thuiller, and F. Courchamp. 2014. Vulnerability of biodiversity hotspots to global change. *Global Ecology and Biogeography* 23: 1376–1386. doi:10.1111/geb.12228.

Biemans, H., L. H. Speelman, F. Ludwig, E. J. Moors, A. J. Wiltshire, P. Kumar, D. Gerten, and P. Kabat. 2013. Future water resources for food production in five South Asian river basins and potential for adaptation - A modeling study. *Science of the Total Environment* 468–469. Elsevier B.V.: S117–S131. doi:10.1016/j.scitotenv.2013.05.092.

Bourne, A., S. Holness, P. Holden, S. Scorgie, C. I. Donatti, and G. Midgley. 2016. A socio-ecological approach for identifying and contextualising spatial ecosystem-based adaptation priorities at the sub-national level. *PLoS ONE* 11: 1–21. doi:10.1371/journal.pone.0155235.

Brito, D., D. O. Moreira, B. R. Coutinho, and M. Oprea. 2012. Ill nature: Disease hotspots as threats to biodiversity. *Journal for Nature Conservation* 20. Elsevier GmbH.: 72–75. doi:10.1016/j.jnc.2011.10.003.

Busby, J. W., K. H. Cook, E. K. Vizy, T. G. Smith, and M. Bekalo. 2014. Identifying hot spots of security vulnerability associated with climate change in Africa. *Climatic Change* 124: 717–731. doi:10.1007/s10584-014-1142-z.

Busby, J. W., T. G. Smith, and N. Krishnan. 2014. Climate security vulnerability in Africa mapping 3.01. *Political Geography* 43. Elsevier Ltd: 51–67. doi:10.1016/j.polgeo.2014.10.005.

Butler, J. R. A., T. Skewes, D. Mitchell, M. Pontio, and T. Hills. 2014. Stakeholder perceptions of ecosystem service declines in Milne Bay, Papua New Guinea: Is human population a more critical driver than climate change? *Marine Policy* 46. Elsevier: 1–13. doi:10.1016/j.marpol.2013.12.011.

Cañadas, E. M., G. Fenu, J. Peñas, J. Lorite, E. Mattana, and G. Bacchetta. 2014. Hotspots within hotspots: Endemic plant richness, environmental drivers, and implications for conservation. *Biological Conservation* 170: 282–291. doi:10.1016/j.biocon.2013.12.007.

Carvalho, A. F., and M. A. Del Lama. 2015. Predicting priority areas for conservation from historical climate modelling: stingless bees from Atlantic Forest hotspot as a case study. *Journal of Insect Conservation* 19: 581–587. doi:10.1007/s10841-015-9780-7.

Cochrane, L., G. Cundill, E. Ludi, M. New, R. J. Nicholls, P. Wester, B. Cantin, K. S. Murali, et al. 2017. A reflection on collaborative adaptation research in Africa and Asia. *Regional Environmental Change* 17. Springer Berlin Heidelberg: 1–9. doi:10.1007/s10113-017-1140-6.

Davies, P. M. 2010. Climate change implications for river restoration in global biodiversity hotspots. *Restoration Ecology* 18: 261–268. doi:10.1111/j.1526-100X.2009.00648.x.

Davis, J., A. P. O'Grady, A. Dale, A. H. Arthington, P. A. Gell, P. D. Driver, N. Bond, M. Casanova, et al. 2015. When trends intersect: The challenge of protecting freshwater ecosystems under multiple land use and hydrological intensification scenarios. *Science of the Total Environment* 534. Elsevier B.V.: 65–78. doi:10.1016/j.scitotenv.2015.03.127.

de Sherbinin, A. 2014. Climate change hotspots mapping: What have we learned? *Climatic Change* 123: 23–37. doi:10.1007/s10584-013-0900-7.

de Sherbinin, A., T. Chai-Onn, M. Jaiteh, V. Mara, L. Pistolesi, E. Schnarr, and S. Trzaska. 2015. Data Integration for Climate Vulnerability Mapping in West Africa. *ISPRS International Journal of Geo-Information* 4: 2561–2582. doi:10.3390/ijgi4042561.

Deregibus, D., M. L. Quartino, K. Zacher, G. L. Campana, and D. K. A. Barnes. 2017. Understanding the link between sea ice, ice scour and Antarctic benthic biodiversity–the need for cross-station and international collaboration. *Polar Record* 53: 1–10. doi:10.1017/S0032247416000875.

Descombes, P., M. S. Wisz, F. Leprieur, V. Parravicini, C. Heine, S. M. Olsen, D. Swingedouw, M. Kulbicki, et al. 2015. Forecasted coral reef decline in marine biodiversity hotspots under climate change. *Global Change Biology* 21: 2479–2487. doi:10.1111/gcb.12868.

Diffenbaugh, N. S., and F. Giorgi. 2012. Climate change hotspots in the CMIP5 global climate model ensemble. *Climatic Change* 114: 813–822. doi:10.1007/s10584-012-0570-x.

Diffenbaugh, N. S., F. Giorgi, and J. S. Pal. 2008. Climate change hotspots in the United States. *Geophysical Research Letters* 35: 1–5. doi:10.1029/2008GL035075.

Dubey, S. K., R. K. Trivedi, B. K. Chand, B. Mandal, and S. K. Rout. 2017. Farmers? perceptions of climate change, impacts on freshwater aquaculture and adaptation strategies in climatic change hotspots: A case of the Indian Sundarban delta. *Environmental Development* 21. Elsevier Ltd: 38–51. doi:10.1016/j.envdev.2016.12.002.

Emrich, C. T., and S. L. Cutter. 2011. Social Vulnerability to Climate-Sensitive Hazards in the Southern United States. *Weather, Climate, and Society* 3: 193–208. doi:10.1175/2011WCAS1092.1.

Feeley, K. J., and M. R. Silman. 2010. Land-use and climate change effects on population size and extinction risk of Andean plants. *Global Change Biology* 16: 3215–3222. doi:10.1111/j.1365-2486.2010.02197.x.

Fern??ndez, I. C., and N. S. Morales. 2016. A spatial multicriteria decision analysis for selecting priority sites for plant species restoration: a case study from the Chilean biodiversity hotspot. *Restoration Ecology* 24: 599–608. doi:10.1111/rec.12354.

Figueredo, C. C., G. von Rückert, and A. Giani. 2016. The necessity of management in a lake of the Atlantic Forest biodiversity hotspot: nitrogen levels connected to a persistent bloom of Cylindrospermopsis raciborskii. *Acta Botanica Brasilica* 30: 0–0. doi:10.1590/0102-33062015abb0334.

Filho, J. P. D., D. M. Lapola, R. R. Torres, and M. C. Lemos. 2016. Socio-climatic hotspots in Brazil: how do changes driven by the new set of IPCC climatic projections affect their relevance for policy? *Climatic Change* 136. Climatic Change: 413–425. doi:10.1007/s10584-016-1635-z.

Foufoula-georgiou, E. F. I. 2013. A vision for a coordinated international effort on delta sustainability. *Deltas: Landforms, Ecosystems and Human Activities* 358: 3–11.

Fraser, E. D. G., E. Simelton, M. Termansen, S. N. Gosling, and A. South. 2013. " Vulnerability hotspots" : Integrating socio-economic and hydrological models to identify where cereal production may decline in the future due to climate change induced drought. *Agricultural and Forest Meteorology* 170. Elsevier B.V.: 195–205. doi:10.1016/j.agrformet.2012.04.008.

Freudenberger, L., P. Hobson, M. Schluck, S. Kreft, K. Vohland, H. Sommer, S. Reichle, C. Nowicki, et al. 2013. Nature conservation: Priority-setting needs a global change. *Biodiversity and Conservation* 22: 1255–1281. doi:10.1007/s10531-012-0428-6.

Giorgi, F. 2006. Climate change hot-spots. *Geophysical Research Letters* 33: 1–4. doi:10.1029/2006GL025734.

Gomez, C., M. Mangeas, T. Curt, T. Ibanez, J. Munzinger, P. Dumas, A. Jérémy, M. Despinoy, et al. 2015. Wildfire risk for main vegetation units in a biodiversity hotspot: Modeling approach in New Caledonia, South Pacific. *Ecology and Evolution* 5: 377–390. doi:10.1002/ece3.1317.

Gormley, K. S. G., A. D. Hull, J. S. Porter, M. C. Bell, and W. G. Sanderson. 2015. Adaptive management, international co-operation and planning for marine conservation hotspots in a changing climate. *Marine Policy* 53. Elsevier: 5466. doi:10.1016/j.marpol.2014.11.017.

Goulding, W., P. T. Moss, and C. A. McAlpine. 2016. Cascading effects of cyclones on the biodiversity of Southwest Pacific islands. *Biological Conservation* 193. Elsevier Ltd: 143–152. doi:10.1016/j.biocon.2015.11.022.

Graham, T. L., H. D. Matthews, and S. E. Turner. 2016. A Global-Scale Evaluation of Primate Exposure and Vulnerability to Climate Change. *International Journal of Primatology* 37. International Journal of Primatology: 158–174. doi:10.1007/s10764-016-9890-4.

Grecian, W. J., M. J. Witt, M. J. Attrill, S. Bearhop, P. H. Becker, C. Egevang, R. W. Furness, B. J. Godley, et al. 2016. Seabird diversity hotspot linked to ocean productivity in the Canary Current Large Marine Ecosystem. *Biology Letters* 12: 20160024. doi:10.1098/rsbl.2016.0024.

Hagenlocher, M., S. Lang, D. Holbling, D. Tiede, and S. Kienberger. 2014. Modeling hotspots of climate change in the sahel using object-based regionalization of multidimensional gridded datasets. *IEEE Journal of Selected Topics in Applied Earth Observations and Remote Sensing* 7: 229–234. doi:10.1109/JSTARS.2013.2259579.

Hagerman, S. M. 2016. Governing adaptation across scales: Hotspots and hesitancy in Pacific Northwest forests. *Land Use Policy* 52. Elsevier Ltd: 306–315. doi:10.1016/j.landusepol.2015.12.034.

Hannah, L., M. Ikegami, D. G. Hole, C. Seo, S. H. M. Butchart, A. T. Peterson, and P. R. Roehrdanz. 2013. Global Climate Change Adaptation Priorities for Biodiversity and Food Security. *PLoS ONE* 8. doi:10.1371/journal.pone.0072590.

Hare, W. L., W. Cramer, M. Schaeffer, A. Battaglini, and C. C. Jaeger. 2011. Climate hotspots: Key vulnerable regions, climate change and limits to warming. *Regional Environmental Change* 11: 1–13. doi:10.1007/s10113-010-0195-4.

Hartter, J., M. D. Stampone, S. J. Ryan, K. Kirner, C. A. Chapman, and A. Goldman. 2012. Patterns and perceptions of climate change in a biodiversity conservation hotspot. *PLoS ONE* 7. doi:10.1371/journal.pone.0032408.

Hartter, J., N. Dowhaniuk, C. A. MacKenzie, S. J. Ryan, J. E. Diem, M. W. Palace, and C. A. Chapman. 2016. Perceptions of risk in communities near parks in an African biodiversity hotspot. *Ambio* 45. Springer Netherlands: 692–705. doi:10.1007/s13280-016-0775-8.

He, T., H. D'Agui, S. L. Lim, N. J. Enright, and Y. Luo. 2016. Evolutionary potential and adaptation of Banksia attenuata (Proteaceae) to climate and fire regime in southwestern Australia, a global biodiversity hotspot. *Scientific Reports* 6. Nature Publishing Group: 26315. doi:10.1038/srep26315.

Hermans-Neumann, K., J. Priess, and M. Herold. 2017. Human migration, climate variability, and land degradation: hotspots of socio-ecological pressure in Ethiopia. *Regional Environmental Change* 17. Springer Berlin Heidelberg: 1–14. doi:10.1007/s10113-017-1108-6.

Hirschfeld, M., D. C. Blackburn, T. M. Doherty-Bone, L. N. Gonwouo, S. Ghose, and M.-O. Rödel. 2016. Dramatic Declines of Montane Frogs in a Central African Biodiversity Hotspot. *Plos One* 11: e0155129. doi:10.1371/journal.pone.0155129.

Hl??sny, T., J. Trombik, L. Dobor, Z. Barcza, and I. Barka. 2016. Future climate of the Carpathians: climate change hot-spots and implications for ecosystems. *Regional Environmental Change* 16: 1495–1506. doi:10.1007/s10113-015-0890-2.

Hobday, A. J., and G. T. Pecl. 2014. Identification of global marine hotspots: sentinels for change and vanguards for adaptation action. *Reviews in Fish Biology and Fisheries* 24: 415–425. doi:10.1007/s11160-013-9326-6.

Hobday, A. J., K. Cochrane, N. Downey-Breedt, J. Howard, S. Aswani, V. Byfield, G. Duggan, E. Duna, et al. 2016. Planning adaptation to climate change in fast-warming marine regions with seafood-dependent coastal communities. *Reviews in Fish Biology and Fisheries* 26: 249–264. doi:10.1007/s11160-016-9419-0.

Hua, W. J., H. S. Chen, S. G. Zhu, S. L. Sun, M. Yu, and L. M. Zhou. 2013. Hotspots of the sensitivity of the land surface hydrological cycle to climate change. *Chinese Science Bulletin* 58: 3682–3688. doi:10.1007/s11434-013-5846-7.

Hugo, G. 2011. Future demographic change and its interactions with migration and climate change. *Global Environmental Change* 21. Elsevier Ltd: S21–S33. doi:10.1016/j.gloenvcha.2011.09.008.

Iwamura, T., A. Guisan, K. A. Wilson, and H. P. Possingham. 2013. How robust are global conservation priorities to climate change? *Global Environmental Change* 23. Elsevier Ltd: 1277–1284. doi:10.1016/j.gloenvcha.2013.07.016.

Jantz, S. M., B. Barker, T. M. Brooks, L. P. Chini, Q. Huang, R. M. Moore, J. Noel, and G. C. Hurtt. 2015. Future habitat loss and extinctions driven by land-use change in biodiversity hotspots under four scenarios of climate-change mitigation. *Conservation Biology* 29: 1122–1131. doi:10.1111/cobi.12549.

Kano, Y., D. Dudgeon, S. Nam, H. Samejima, K. Watanabe, C. Grudpan, J. Grudpan, W. Magtoon, et al. 2016. Impacts of dams and global warming on fish biodiversity in the Indo-Burma hotspot. *PLoS ONE* 11: 1–21. doi:10.1371/journal.pone.0160151.

Kelly, P., L. Clementson, C. Davies, S. Corney, and K. Swadling. 2016. Zooplankton responses to increasing sea surface temperatures in the southeastern Australia global marine hotspot. *Estuarine, Coastal and Shelf Science* 180. Elsevier Ltd: 242–257. doi:10.1016/j.ecss.2016.07.019.

Kerr, R., and R. Kerr. 2008. Climate Change Hot Spots Mapped Across the United States. *Science* 321: 909. doi:10.1126/science.321.5891.909.

Khamis, K., D. M. Hannah, M. H. Clarvis, L. E. Brown, E. Castella, and A. M. Milner. 2014. Alpine aquatic ecosystem conservation policy in a changing climate. *Environmental Science and Policy* 43. Elsevier Ltd: 39–55. doi:10.1016/j.envsci.2013.10.004.

Kilroy, G. 2015. A review of the biophysical impacts of climate change in three hotspot regions in Africa and Asia. *Regional Environmental Change* 15. Springer Berlin Heidelberg: 771–782. doi:10.1007/s10113-014-0709-6.

Kostoski, G., C. Albrecht, S. Trajanovski, and T. Wilke. 2010. A freshwater biodiversity hotspot under pressure - Assessing threats and identifying conservation needs for ancient Lake Ohrid. *Biogeosciences* 7: 3999–4015. doi:10.5194/bg-7-3999-2010.

Kovats, R. S. 2002. Clinical review Hotspots in climate change and human health 325. doi:10.1136/bmj.325.7372.1094.

Lah, R. A., K. Benkendorff, and D. Bucher. 2017. Thermal tolerance and preference of exploited turbinid snails near their range limit in a global warming hotspot. *Journal of Thermal Biology* 64: 100–108. doi:10.1016/j.jtherbio.2017.01.008.

LEE, A. T. K., and P. BARNARD. 2016. Endemic birds of the Fynbos biome: a conservation assessment and impacts of climate change. *Bird Conservation International* 26: 52–68. doi:10.1017/S0959270914000537.

Li, X., X. Liu, F. Kraus, R. Tingley, and Y. Li. 2016. Risk of biological invasions is concentrated in biodiversity hotspots. *Frontiers in Ecology and the Environment* 14: 411–417. doi:10.1002/fee.1321.

Liu, H., C. L. Feng, Y. B. Luo, B. S. Chen, Z. S. Wang, and H. Y. Gu. 2010. Potential challenges of climate change to orchid conservation in a Wild Orchid Hotspot in Southwestern China. *Botanical Review* 76: 174–192. doi:10.1007/s12229-010-9044-x.

Liu, J., S. Fritz, C. F. A. van Wesenbeeck, M. Fuchs, L. You, M. Obersteiner, and H. Yang. 2008. A spatially explicit assessment of current and future hotspots of hunger in Sub-Saharan Africa in the context of global change. *Global and Planetary Change* 64. Elsevier B.V.: 222–235. doi:10.1016/j.gloplacha.2008.09.007.

Loyola, R. D., P. Lemes, J. C. Nabout, J. Trindade-Filho, M. D. Sagnori, R. Dobrovolski, and J. A. F. Diniz-Filho. 2013. A straightforward conceptual approach for evaluating spatial conservation priorities under climate change. *Biodiversity and Conservation* 22: 483–495. doi:10.1007/s10531-012-0424-x.

Lutz, D. A., R. L. Powell, and M. R. Silman. 2013. Four Decades of Andean Timberline Migration and Implications for Biodiversity Loss with Climate Change. *PLoS ONE* 8: e74496. doi:10.1371/journal.pone.0074496.

MacCallum, D., J. Byrne, and W. Steele. 2014. Whither justice? An analysis of local climate change responses from South East Queensland, Australia. *Environment and Planning C: Government and Policy* 32: 70–92. doi:10.1068/c11295.

Mairal, M., I. Sanmartín, A. Herrero, L. Pokorny, P. Vargas, J. J. Aldasoro, and M. Alarcón. 2017. Geographic barriers and Pleistocene climate change shaped patterns of genetic variation in the Eastern Afromontane biodiversity hotspot. *Scientific Reports* 7. Nature Publishing Group: 45749. doi:10.1038/srep45749.

Mantyka-Pringle, C. S., P. Visconti, M. Di Marco, T. G. Martin, C. Rondinini, and J. R. Rhodes. 2015. Climate change modifies risk of global biodiversity loss due to land-cover change. *Biological Conservation* 187. Elsevier Ltd: 103–111. doi:10.1016/j.biocon.2015.04.016.

Marques, M. C. M., M. D. Swaine, and D. Liebsch. 2011. Diversity distribution and floristic differentiation of the coastal lowland vegetation: Implications for the conservation of the Brazilian Atlantic Forest. *Biodiversity and Conservation* 20: 153–168. doi:10.1007/s10531-010-9952-4.

Milne, R., S. J. Cunningham, A. T. K. Lee, and B. Smit. 2015. The role of thermal physiology in recent declines of birds in a biodiversity hotspot. *Conservation Physiology* 3: 1–17. doi:10.1093/conphys/cov048.

Molina-Venegas, R., A. Aparicio, S. Lavergne, and J. Arroyo. 2017. Climatic and topographical correlates of plant palaeo- and neoendemism in a Mediterranean biodiversity hotspot. *Annals of Botany* 119: 229–238. doi:10.1093/aob/mcw093.

Moura, M. R., A. J. Arg??lo, and H. C. Costa. 2017. Historical and contemporary correlates of snake biogeographical subregions in the Atlantic Forest hotspot. *Journal of Biogeography* 44: 640–650. doi:10.1111/jbi.12900.

Müller, C., K. Waha, A. Bondeau, and J. Heinke. 2014. Hotspots of climate change impacts in sub-Saharan Africa and implications for adaptation and development. *Global Change Biology* 20: 2505–2517. doi:10.1111/gcb.12586.

Musinguzi, L., J. Efitre, K. Odongkara, R. Ogutu-Ohwayo, F. Muyodi, V. Natugonza, M. Olokotum, S. Namboowa, et al. 2016. Fishers??? perceptions of climate change, impacts on their livelihoods and adaptation strategies in environmental change hotspots: a case of Lake Wamala, Uganda. *Environment, Development and Sustainability* 18. Springer Netherlands: 1255–1273. doi:10.1007/s10668-015-9690-6.

Nakamura, H., A. Isobe, S. Minobe, H. Mitsudera, M. Nonaka, and T. Suga. 2015. "Hot Spots" in the climate system—new developments in the extratropical ocean–atmosphere interaction research: a short review and an introduction. *Journal of Oceanography* 71. Springer Japan: 463–467. doi:10.1007/s10872-015-0321-5.

Newton, A., and J. Weichselgartner. 2014. Hotspots of coastal vulnerability: A DPSIR analysis to find societal pathways and responses. *Estuarine, Coastal and Shelf Science* 140. Elsevier Ltd: 123–133. doi:10.1016/j.ecss.2013.10.010.

Nguyen, T. T. X., and C. D. Woodroffe. 2016. Assessing relative vulnerability to sea-level rise in the western part of the Mekong River Delta in Vietnam. *Sustainability Science* 11. Springer Japan: 645–659. doi:10.1007/s11625-015-0336-2.

O'Farrell, P. J., B. Reyers, D. C. Le Maitre, S. J. Milton, B. Egoh, A. Maherry, C. Colvin, D. Atkinson, et al. 2010. Multi-functional landscapes in semi arid environments: Implications for biodiversity and ecosystem services. *Landscape Ecology* 25: 1231–1246. doi:10.1007/s10980-010-9495-9.

Paulot, F., D. J. Jacob, and D. K. Henze. 2013. Sources and processes contributing to nitrogen deposition in biodiversity hotspots worldwide. *Environ. Sci. Technol.*: 1–24.

Piontek, F., C. Müller, T. A. M. Pugh, D. B. Clark, D. Deryng, J. Elliott, F. de J. Colón González, M. Flörke, et al. 2014. Multisectoral climate impact hotspots in a warming world. *Proceedings of the National Academy of Sciences* 111: 3233–3238. doi:10.1073/pnas.1222471110.

Platts, P. J., R. E. Gereau, N. D. Burgess, and R. Marchant. 2013. Spatial heterogeneity of climate change in an Afromontane centre of endemism. *Ecography* 36: 518–530. doi:10.1111/j.1600-0587.2012.07805.x.

Popova, E., A. Yool, V. Byfield, K. Cochrane, A. C. Coward, S. S. Salim, M. A. Gasalla, S. A. Henson, et al. 2016. From global to regional and back again: Common climate stressors of marine ecosystems relevant for adaptation across five ocean warming hotspots. *Global Change Biology* 22: 2038–2053. doi:10.1111/gcb.13247.

Post, E., J. Brodie, M. Hebblewhite, A. D. Anders, J. A. K. Maier, and C. C. Wilmers. 2009. Global Population Dynamics and Hot Spots of Response to Climate Change. *BioScience* 59: 489–497. doi:10.1525/bio.2009.59.6.7.

Ramesh, R., Z. Chen, V. Cummins, J. Day, C. D'Elia, B. Dennison, D. L. Forbes, B. Glaeser, et al. 2015. Land-Ocean Interactions in the Coastal Zone: Past, present & future. *Anthropocene* 12. Elsevier B.V.: 85–98. doi:10.1016/j.ancene.2016.01.005.

Ramírez, F., I. Afán, L. S. Davis, and A. Chiaradia. 2017. Climate impacts on global hot spots of marine biodiversity. *Science Advances* 3: e1601198. doi:10.1126/sciadv.1601198.

Roberts, C. M. 2002. Marine Biodiversity Hotspots and Conservation Priorities for Tropical Reefs. *Science* 295: 1280–1284. doi:10.1126/science.1067728.

Schierenbeck, K. A. 2017. Population-level genetic variation and climate change in a biodiversity hotspot. *Annals of Botany* 119: 215–228. doi:10.1093/aob/mcw214.

Sharma, R. 2012. Impacts on Human Health of Climate and Land Use Change in the Hindu Kush–Himalayan Region. *Mountain Research and Development* 32: 480–486. doi:10.1659/MRD-JOURNAL-D-12-00068.1.

Sheng, L., S. Liu, and H. Liu. 2010. Climate-induced hotspots in surface energy fluxes from 1948 to 2000. *Environmental Research Letters* 5: 14001. doi:10.1088/1748-9326/5/1/014001.

Shukla, R., K. Sachdeva, and P. K. Joshi. 2016. Inherent vulnerability of agricultural communities in Himalaya: A village-level hotspot analysis in the Uttarakhand state of India. *Applied Geography* 74. Elsevier Ltd: 182–198. doi:10.1016/j.apgeog.2016.07.013.

Slingsby, J. A., C. Merow, M. Aiello-Lammens, N. Allsopp, S. Hall, H. Kilroy Mollmann, R. Turner, A. M. Wilson, et al. 2017. Intensifying postfire weather and biological invasion drive species loss in a Mediterranean-type biodiversity hotspot. *Proceedings of the National Academy of Sciences* 114: 201619014. doi:10.1073/pnas.1619014114.

Stork, N. E., and J. C. Habel. 2014. Can biodiversity hotspots protect more than tropical forest plants and vertebrates? *Journal of Biogeography* 41: 421–428. doi:10.1111/jbi.12223.

Tchebakova, N. M., E. I. Parfenova, and A. J. Soja. 2011. Climate change and climate-induced hot spots in forest shifts in central Siberia from observed data. *Regional Environmental Change* 11: 817–827. doi:10.1007/s10113-011-0210-4.

Thom, D., W. Rammer, T. Dirnb??ck, J. M??ller, J. Kobler, K. Katzensteiner, N. Helm, and R. Seidl. 2017. The impacts of climate change and disturbance on spatio-temporal trajectories of biodiversity in a temperate forest landscape. *Journal of Applied Ecology* 54: 28–38. doi:10.1111/1365-2664.12644.

Timonen, J., L. Gustafsson, J. S. Kotiaho, and M. Mönkkönen. 2011. Hotspots in cold climate: Conservation value of woodland key habitats in boreal forests. *Biological Conservation* 144. Elsevier Ltd: 2061–2067. doi:10.1016/j.biocon.2011.02.016.

Torres, R. R., and J. A. Marengo. 2014. Climate change hotspots over South America: From CMIP3 to CMIP5 multi-model datasets. *Theoretical and Applied Climatology* 117: 579–587. doi:10.1007/s00704-013-1030-x.

Tran, D. X., F. Pla, P. Latorre-Carmona, S. W. Myint, M. Caetano, and H. V. Kieu. 2017. Characterizing the relationship between land use land cover change and land surface temperature. *ISPRS Journal of Photogrammetry and Remote Sensing* 124. International Society for Photogrammetry and Remote Sensing, Inc. (ISPRS): 119–132. doi:10.1016/j.isprsjprs.2017.01.001.

Trape, S. 2016. A new cichlid fish in the Sahara: The Ounianga Serir lakes (Chad), a biodiversity hotspot in the desert. *Comptes Rendus Biologies* in press: 529–536. doi:10.1016/j.crvi.2016.08.003.

Tucker, J., M. Daoud, N. Oates, R. Few, D. Conway, S. Mtisi, and S. Matheson. 2015. Social vulnerability in three high-poverty climate change hot spots: What does the climate change literature tell us? *Regional Environmental Change* 15. Springer Berlin Heidelberg: 783–800. doi:10.1007/s10113-014-0741-6.

Turco, M., E. Palazzi, J. von Hardenberg, and A. Provenzale. 2015. Observed climate change. *Geophysical Research Letters* 42: 3521–3528. doi:10.1002/2015GL063891.Received.

Vermaat, J. E., and M. A. Eleveld. 2013. Divergent options to cope with vulnerability in subsiding deltas. *Climatic Change* 117: 31–39. doi:10.1007/s10584-012-0532-3.

Wan, J. Z., C. J. Wang, and F. H. Yu. 2016. Risk hotspots for terrestrial plant invaders under climate change at the global scale. *Environmental Earth Sciences* 75. Springer Berlin Heidelberg: 1–8. doi:10.1007/s12665-016-5826-8.

Wang, J., Y. Lu, F. Wang, and R.-H. Zhang. 2017. Surface Current in “Hotspot” Serves as a New and Effective Precursor for El Niño Prediction. *Scientific Reports* 7. Springer US: 166. doi:10.1038/s41598-017-00244-2.

Ward, E. J., J. E. Jannot, Y. W. Lee, K. Ono, A. O. Shelton, and J. T. Thorson. 2015. Using spatiotemporal species distribution models to identify temporally evolving hotspots of species co-occurrence. *Ecological Applications* 25: 2198–2209. doi:10.1890/15-0051.1.

Wardell-Johnson, G., A. Wardell-Johnson, K. Bradby, T. Robinson, P. W. Bateman, K. Williams, A. Keesing, K. Braun, et al. 2016. Application of a Gondwanan perspective to restore ecological integrity in the south-western Australian global biodiversity hotspot. *Restoration Ecology* 24: 805–815. doi:10.1111/rec.12372.

Wernberg, T., B. D. Russell, P. J. Moore, S. D. Ling, D. A. Smale, A. Campbell, M. A. Coleman, P. D. Steinberg, et al. 2011. Impacts of climate change in a global hotspot for temperate marine biodiversity and ocean warming. *Journal of Experimental Marine Biology and Ecology* 400. Elsevier B.V.: 7–16. doi:10.1016/j.jembe.2011.02.021.

Wetzel, F. T., W. D. Kissling, H. Beissmann, and D. J. Penn. 2012. Future climate change driven sea-level rise: Secondary consequences from human displacement for island biodiversity. *Global Change Biology* 18: 2707–2719. doi:10.1111/j.1365-2486.2012.02736.x.

Wramneby, A., B. Smith, and P. Samuelsson. 2010. Hot spots of vegetation-climate feedbacks under future greenhouse forcing in Europe. *Journal of Geophysical Research Atmospheres* 115: 1–12. doi:10.1029/2010JD014307.

Wu, Y., S. G. DuBay, R. K. Colwell, J. Ran, and F. Lei. 2017. Mobile hotspots and refugia of avian diversity in the mountains of south-west China under past and contemporary global climate change. *Journal of Biogeography* 44: 615–626. doi:10.1111/jbi.12862.

Xu, J., and R. E. Grumbine. 2014. Building ecosystem resilience for climate change adaptation in the Asian highlands. *Wiley Interdisciplinary Reviews: Climate Change* 5: 709–718. doi:10.1002/wcc.302.

Ying, X. U. 2009. Regional Variability of Climate Change Hot-Spots in East Asia. *Advances* 26: 783–792. doi:10.1007/s00376-009-9034-2.1.Introduction.

Zamborlini Saiter, F., J. L. Brown, W. W. Thomas, A. T. de Oliveira-Filho, and A. C. Carnaval. 2016. Environmental correlates of floristic regions and plant turnover in the Atlantic Forest hotspot. *Journal of Biogeography* 43: 2322–2331. doi:10.1111/jbi.12774.

CARIAA Research outputs included in synthesis

Cochrane, L., G. Cundill, E. Ludi, M. New, R. J. Nicholls, P. Wester, B. Cantin, K. S. Murali, et al. 2017. A reflection on collaborative adaptation research in Africa and Asia. *Regional Environmental Change* 17. Springer Berlin Heidelberg: 1–9. doi:10.1007/s10113-017-1140-6.

Few, R., D. Morchain, D. Spear, A. Mensah, and R. Bendapudi. 2017. Transformation, adaptation and development: relating concepts to practice. *Palgrave Communications* 3. Nature Publishing Group: 17092. doi:10.1057/palcomms.2017.92.

Bettini, G., S. L. Nash, and G. Gioli. 2016. One step forward, two steps back? The fading contours of (in)justice in competing discourses on climate migration. *Geographical Journal*. doi:10.1111/geoj.12192.

Rao, N., E. T. Lawson, W. N. Raditloaneng, D. Solomon, and M. N. Angula. 2017. Gendered vulnerabilities to climate change: insights from the semi-arid regions of Africa and Asia. *Climate and Development* 0. Taylor & Francis: 1–13. doi:10.1080/17565529.2017.1372266.

Singh, C., J. Daron, A. Bazaz, G. Ziervogel, D. Spear, J. Krishnaswamy, M. Zaroug, and E. Kituyi. 2017. The utility of weather and climate information for adaptation decision-making: current uses and future prospects in Africa and India. *Climate and Development* 0. Taylor & Francis: 1–17. doi:10.1080/17565529.2017.1318744.

Toure, M. A., M. L. Ndiaye, V. B. Traore, G. Faye, B. Cisse, A. Ndiaye, and C. T. Wade. 2016. Using of Landsat Images for Land Use Changes Detection in the Ecosystem : A Case Study of the Senegal River Delta.

Binder, C. R., and P. Bots. 2013. Comparison of Frameworks for Analyzing Social- ecological Systems Comparison of Frameworks for Analyzing Social-ecological Systems 18. doi:10.5751/ES-05551-180426.

Kastner, M., A. C. Tricco, C. Soobiah, E. Lillie, L. Perrier, T. Horsley, V. Welch, E. Cogo, et al. 2012. What is the most appropriate knowledge synthesis method to conduct a review? Protocol for a scoping review. *BMC Medical Research Methodology* 12: 114. doi:10.1186/1471-2288-12-114.

Kraaijenbrink, P. D. A., M. F. P. Bierkens, A. F. Lutz, and W. W. Immerzeel. 2017. Impact of a global temperature rise of 1.5 degrees Celsius on Asia’s glaciers. *Nature* 549. Nature Publishing Group: 257–260. doi:10.1038/nature23878.

Saha, D. 2016. Agricultural Adaptation Practices in Coastal Bangladesh : Response to Climate Change Impacts Agricultural Adaptation Practices in Coastal Bangladesh : Response to Climate Change Impacts.

Ahmed, A., E. T. Lawson, A. Mensah, C. Gordon, and J. Padgham. 2016. Adaptation to climate change or non-climatic stressors in semi-arid regions? Evidence of gender differentiation in three agrarian districts of Ghana. *Environmental Development* 20. Elsevier: 45–58. doi:10.1016/j.envdev.2016.08.002.

Adhikari, L., A. Hussain, and G. Rasul. 2017. Tapping the potential of neglected and underutilized food crops for sustainable nutrition security in the mountains of Pakistan and Nepal. *Sustainability (Switzerland)* 9. doi:10.3390/su9020291.

Crawford, A., and A. Terton. 2015. Review of Current and Planned Adaptation Action in Tanzania - Draft.

Karpouzoglou, T., and S. Vij. 2017. Waterscape: a perspective for understanding the contested geography of water. *Wiley Interdisciplinary Reviews: Water* 4: e1210. doi:10.1002/wat2.1210.

Thomas, R., and V. Duraisamy. 2016. Hydrogeological delineation of groundwater vulnerability to droughts in semi-arid areas of western Ahmednagar district. *Egyptian Journal of Remote Sensing and Space Science*. National Authority for Remote Sensing and Space Sciences. doi:10.1016/j.ejrs.2016.11.008.

Lazar, A. N., R. J. Nicholls, A. Payo, H. Adams, C. Mortreux, N. Suckall, K. Vincent, H. Sugata, et al. Working Paper A method to assess migration and adaptation in deltas : A preliminary fast-track assessment.

Arfanuzzaman, M., and M. Abu Syed. 2017. Water demand and ecosystem nexus in the transboundary river basin: a zero-sum game. *Environment, Development and Sustainability*. Springer Netherlands: 1–12. doi:10.1007/s10668-017-9915-y.

Szabo, S., R. J. Nicholls, B. Neumann, F. G. Renaud, Z. Matthews, Z. Sebesvari, A. AghaKouchak, R. Bales, et al. 2016. Making SDGs work for climate change hotspots. *Environment* 58: 24–33. doi:10.1080/00139157.2016.1209016.

Lazzaroni, S., and N. Wagner. 2016. Misfortunes never come singly: Structural change, multiple shocks and child malnutrition in rural Senegal. *Economics and Human Biology* 23. Elsevier B.V.: 246–262. doi:10.1016/j.ehb.2016.10.006.

Vincent, K., T. Cull, and K. Integrated. 2016. Scoping report on adaptation finance initiatives in Bangladesh , Ghana and India: 51.

Welch, A. C., R. J. Nicholls, and A. N. Lázár. 2017. Evolving deltas : Coevolution with engineered interventions. doi:10.1525/elementa.128.

Lutz, A. F., W. W. Immerzeel, P. D. A. Kraaijenbrink, A. B. Shrestha, and M. F. P. Bierkens. 2016. Climate change impacts on the upper indus hydrology: Sources, shifts and extremes. *PLoS ONE* 11: 1–33. doi:10.1371/journal.pone.0165630.

Thapa, B., C. Scott, P. Wester, and R. Varady. 2016. Towards characterizing the adaptive capacity of farmer-managed irrigation systems: learnings from Nepal. *Current Opinion in Environmental Sustainability* 21. Elsevier B.V.: 37–44. doi:10.1016/j.cosust.2016.10.005.

Bedelian, C., and J. O. Ogutu. 2017. Trade-offs for climate-resilient pastoral livelihoods in wildlife conservancies in the Mara ecosystem, Kenya. *Pastoralism* 7. Pastoralism: 10. doi:10.1186/s13570-017-0085-1.

Patra, J. 2014. *Science – policy interface for disaster risk management in India : toward an enabling*. Vol. 107.

Akber, M. A., M. A. Islam, M. Ahmed, M. M. Rahman, and M. R. Rahman. 2017. Changes of shrimp farming in southwest coastal Bangladesh. *Aquaculture International* 25. Aquaculture International: 1883–1899. doi:10.1007/s10499-017-0159-5.

Biemans, H., C. Siderius, A. Mishra, and B. Ahmad. 2016. Crop-specific seasonal estimates of irrigation-water demand in South Asia. *Hydrology and Earth System Sciences* 20: 1971–1982. doi:10.5194/hess-20-1971-2016.

Lutz, A. F., H. W. ter Maat, H. Biemans, A. B. Shrestha, P. Wester, and W. W. Immerzeel. 2016. Selecting representative climate models for climate change impact studies: an advanced envelope-based selection approach. *International Journal of Climatology* 36: 3988–4005. doi:10.1002/joc.4608.

Collier, E., F. Maussion, L. I. Nicholson, T. Mölg, W. W. Immerzeel, and A. B. G. Bush. 2015. Impact of debris cover on glacier ablation and atmosphere-glacier feedbacks in the Karakoram. *Cryosphere* 9: 1617–1632. doi:10.5194/tc-9-1617-2015.

Morchain, D., G. Prati, F. Kelsey, and L. Ravon. 2015. What if gender became an essential, standard element of Vulnerability Assessments? *Gender and Development* 23: 481–496. doi:10.1080/13552074.2015.1096620.

Rasul, G., and B. Sharma. 2016. The nexus approach to water–energy–food security: an option for adaptation to climate change. *Climate Policy* 16. Taylor & Francis: 682–702. doi:10.1080/14693062.2015.1029865.
